# Utilization of personal protective equipment and associated factors among textile factory workers at Hawassa Town, Southern Ethiopia

**DOI:** 10.1186/s12995-016-0096-7

**Published:** 2016-02-19

**Authors:** Sebsibe Tadesse, Temesgen Kelaye, Yalemzewod Assefa

**Affiliations:** Institute of Public Health, The University of Gondar, Gondar, Ethiopia; Southern Nations Nationalities and People’s Regional State Health Bureau, Hawassa, Ethiopia

**Keywords:** Hazard control, Personal protective equipment, Safety, Textile factory, Workers

## Abstract

**Background:**

Use of personal protective equipment is one of the important measures to safeguard workers from exposure to occupational hazards, especially in developing countries. However, there is a dearth of studies describing personal protective equipment utilization in Ethiopia. The present study has determined the magnitude of personal protective equipment utilization and identified associated factors among textile factory workers at Hawassa Town, southern Ethiopia.

**Methods:**

An institution-based cross-sectional study was conducted among textile factory workers at Hawassa Town, southern Ethiopia from January to March 2014. Stratified sampling followed by simple random sampling techniques was used to select the total of 660 study participants. A pre-tested and structured questionnaire was used to collect data. Multivariate analyses were employed to see the effect of explanatory variables on dependent variable.

**Results:**

The magnitude of personal protective equipment utilization was 82.4 %. Service duration of >10 years [AOR: 0.23, 95 % CI: (0.09, 0.58)], availability of personal protective equipments [AOR: 21.73, 95 % CI: (8.62, 54.79)], shift work [AOR: 2.28, 95 % CI: (1.12, 4.66)], alcohol drinking [AOR: 0.26, 95 % CI: (0.10, 0.66)], and cigarette smoking [AOR: 0.20, 95 % CI: (0.05, 0.78)] were factors significantly associated with use of personal protective equipment.

**Conclusion:**

In this study a relatively higher personal protective equipment utilization rate was reported compared to other studies in developing countries. However, this does not mean that there will be no need for further strengthening the safety programs as there are significant proportion of the workers still does not use all the necessary personal protective equipment during work. Interventions to promote use personal protective equipment should focus on areas, such as service duration, availability of protective equipment, presence of shift work, and control of substance abuse.

## Background

The International Labour Organization has estimated that over 2 million people die every year from work-related accidents and diseases, and over 300 million non-fatal accidents are recorded each year [1]. This translates into more than 6000 deaths and over 800,000 non-fatal accidents every day. As a result of these, there is an estimated economic loss of more than $1.25 trillion each year, which is equivalent to 4.0 % of the world’s Gross Domestic Product. This loss is 4 times higher for developing countries than that of industrialized countries [2, 3]. In many low and middle income countries, such as Ethiopia statistics on occupational accidents and injuries are limited. In cases where such information is available it is largely impaired by underreporting. Considering the differences between countries, it is reasonable to agree that occupational deaths and injuries take a heavy toll among the poor and least protected.

Textile industry is one of labor intensive production and most technologically complex of all industries, and is a place of work where workers are exposed to different safety hazards, like cotton dust, excessive noise, accidents and diseases [4–7]. As result of this, workers, employers and government lost direct and indirect costs related to workplace injuries and illnesses [8]. The direct costs for employers include compensation and treatment costs that has to be paid to the disabled workers, and while the indirect costs include production disturbance costs, lost time of injured worker, time lost by supervisors or executive to follow the injured worker, training costs for new workers. The direct costs for workers include pain and suffering from the injury or illness, loss of income, loss of a job and health-care costs, and while the indirect costs include time lost by family members to care the disabled worker and utmost economic shock and social chaos [9, 10].

Use of personal protective equipment (PPE) is one of the important measures to safeguard workers from exposure to occupational hazards, especially in developing countries where conventional occupational safety control principles remain a challenge to implement [11–13]. Workers use of PPE is affected by socio-demographic, behavioral and work environment factors [14]. In textile factory they use different protective devices at different production sections. For example, they need to wear respirator, gloves, goggle, boot shoes, overall, ear plugs and mask at spinning section, and while reflector and helmet are worn in addition at engineering section.

Policies or measures for delivering health and safety services to factory workers are limited in Ethiopia. This not only limits their access to information and training opportunities but also places the workers at a greater risk to occupational injuries and diseases. Furthermore, literatures show that factory workers lack the knowledge on proper use of protective measures and are least aware of health effects emanating from the activities and materials in their work environments [13, 15, 16].

There is a dearth of studies describing PPE utilization among textile factory workers in most of Subsaharan African countries, like Ethiopia. This paper presents the findings of a study which investigated PPE utilization and associated factors among textile factory workers in southern Ethiopia, a low-income country in East Africa. This study addresses a critical gap in understanding PPE utilization in Ethiopia and contributes to the growing workplace safety research in low-income countries. Such studies may also help in developing evidence-based intervention strategies targeted at promoting workers safety and health.

## Methods

### Study design, area and period, and source population

An institution-based cross-sectional study was conducted to assess the magnitude of PPE utilization and associated factors among textile factory workers at Hawassa Town, southern Ethiopia, from January to March 2014. The town is located at 275 km South of Addis Ababa, the capital city of Ethiopia. During the investigation, there are about 1370 workers in the factory. Of whom, 65.0 % are permanent, 32.3 % temporary, and 2.7 % daily workers [17].

### Participants and data collection

All textile factory workers who directly involved in the process of production at spinning, weaving, finishing, engineering, and garmenting departments were included in the study until the required sample size was obtained. Workers who were absent from work due to reasons other than work-related injuries during the time of data collection were excluded from the study. A pre-tested and structured interview questionnaire was used to collect the data. The questionnaire contained detail information on socio-demographic, behavioral and environmental factors, and utilization of PPE. Except age, all the factors were measured using a categorical scale of measurement.

### Sample size calculation

Epi info version 7 was used to determine the sample size required for this study by taking 1370 total population, 49.3 % expected proportion of sickness absenteeism, 3 % confidence limit, and 95 % confidence level. By adding 10 % non-response rate, the total sample size was 660.

### Sampling procedure

Stratified sampling followed by simple random sampling techniques was used to select the study participants. That is, the manufacturing units were stratified into five departments: spinning, weaving, finishing, engineering, and garment department. Then, the total of 660 samples was proportionally allocated to each department. The participants were drawn from the factory’s list of workers using simple random sampling.

### Data analyses

The training of data collectors and supervisors emphasized issues such as data collection instrument, field methods, inclusion–exclusion criteria, and record keeping. The principal investigator and supervisors coordinated the interview process, spot-checked and reviewed the completed questionnaire on a daily basis to ensure the completeness and consistency of the data collected. The interview questionnaire was pre-tested on 20 workers who work in a factory nearly similar to Hawassa Textile Factory in order to identify potential problem areas, unanticipated interpretations, and cultural objections to any of the questions. Based on the pre-test results the questionnaire was adjusted contextually.

Data entered and cleaned using Epi info version 7 statistical software were analyzed on SPSS version 16. Frequency distribution, mean, standard deviation, and percentage, were used for descriptive analyses. All independent variables were fitted separately into bivariate logistic regression model to evaluate the degree of association with utilization of PPE. Then, variables with a p-value < 0.20 were exported to multivariate logistic regression model to control confounders. The odds ratio (OR) with a 95 % confidence interval (CI) was used to test the statistical significance of variables. The crude OR was used to present the bivariate model outputs while the adjusted OR to the multivariate outputs.

### Operational definitions

#### Utilization of PPE

Use of all the necessary worker-specialized clothing or equipment by workers for protection against health and safety hazards in the workplace [18]. Workers were classified as *those who used PPE* when they were observed wearing of all the PPE that were necessary to be worn during work in a particular working section. The necessarily worn PPE were: 1) a respirator, gloves, eye protector, boot shoes, overall, ear plugs and mask at spinning section, 2) respirator, gloves, eye protector, boot shoes, ear plugs and overall at weaving section, 3) respirator, gloves, mask, ear plugs, boot shoes and overall at finishing section, 4) respirator, gloves, boot shoes, eye protector, overall, reflector, mask and helmet at engineering section, and 5) gloves, boot shoes, mask and overall at garmenting section.

#### Worker

A person who is directly involved in the process of production and has an employment relationship with an employer [19].

#### Marital status

Workers are classified as married and single based on their risk difference to unsafe acts.

#### Ethical considerations

The study protocol was reviewed and approved by the Institutional Review Board of the University of Gondar via the Institute of Public Health. Permission was obtained from Hawassa Textile Factory Administrative Office prior to data collection. Study participants were interviewed after informed written consent was obtained. They were also informed that their participation was voluntary and that they could withdraw from the interview at any time without consequences. The participants were assured that their responses would be treated confidentially through the use of strict coding measures. Finally, safety education was given to workers who reported not utilizing the necessary PPE.

## Results

### Socio-demographic characteristics

A total of 660 workers who directly involved in the process of production participated in the study which makes the response rate 100 %. Of whom 80.8 % were males and 19.2 % females. The mean age with a standard deviation of the workers was 32.9 ± 9.3. More than two-thirds, 68.8 %, of them belonged to the age group of 18-39 years. The majority, 60.5 %, attended secondary and above education. About half, 47.0 %, served for more than 10 years in the factory. The majority, 58.0 %, of the workers were married. Nearly two-thirds, 63.3 %, had a monthly income of less than or equal to Birr 1500 (Table 1).

**Table 1:** Socio-demographic characteristics of textile factory workers at Hawassa Town, southern Ethiopia, 2014

Variables	Number	Percent
Sex		
Male	533	80.8
Female	127	19.2
Age (in years)		
18–25	200	30.3
26–39	254	38.5
> 39	206	31.2
Educational status		
Illiterate	17	2.5
Primary	244	37.0
Secondary	203	30.8
Above secondary	196	29.7
Service duration (in years)		
1–10	350	53.0
> 10	310	47.0
Marital status		
Married	383	58.0
Single	277	42.0
Monthly income (in Birr)		
1–1000	243	36.8
1001–1500	241	36.5
> 1500	176	26.7

### Behavioral characteristics and environmental conditions

The majority, 80.3, 84.1 and 85.9 %, of the workers reported that they did not drink alcohol, chew khat and smoke cigarette, respectively. Three-fourths, 74.5 %, stated that they were satisfied with their job. The majority, 98.6, 93.6 and 62.9 %, informed that they used work guidelines, received safety orientation and attended safety training, respectively. Nearly two-thirds, 61.5 %, complained that there was no safety supervision during their work. Eighty five percent of the workers reported that there was a work shift in the factory. Nearly half, 47.0 %, stated that there was no work rotation. Poor conditions of ventilation and light in the working rooms were testified by 72.3 and 48.9 % of the workers, respectively (Table 2).

**Table 2:** Behavioral characteristics and environmental conditions of workers at Hawassa Textile Factory, southern Ethiopia, 2014

Variables	Number	Percent
Drink alcohol		
Yes	130	19.7
No	530	80.3
Chew khat		
Yes	105	15.9
No	555	84.1
Smoke cigarette		
Yes	93	14.1
No	567	85.9
Satisfied with job		
Yes	492	74.5
No	168	25.5
Used work guideline		
Yes	651	98.6
No	9	1.4
Attended safety training		
Yes	415	62.9
No	245	37.1
Orientation given		
Yes	618	93.6
No	42	6.4
Safety supervision		
Yes	254	38.5
No	406	61.5
Work shift		
Yes	562	85.2
No	98	14.8
Work rotation		
Yes	350	53.0
No	310	47.0
Ventilation		
Good	183	27.7
Poor	477	72.3
Light		
Good	337	51.1
Poor	323	48.9

### Magnitude of PPE utilization

Out of 660 workers 82.4 % were observed wearing of all the necessary PPE during work at the time of interview. Of whom 82.0 % were males and 18.0 % females. The majority, 68.6 %, of them belonged to the age group of 18 to 39 years. Fifty eight percent were married.

The magnitude of PPE utilization was 73.0 % spinning section, 76.0 % weaving section, 87.6 % finishing section, 87.3 % engineering section, and 88.2 % garmenting section (Table 3).

**Table 3:** Magnitude of PPE utilization by working section among textile factory workers in Hawassa Town, southern Ethiopia, 2014

Working section	Number	Percent
Spinning section		
Used	199	73.0
Not used	73	27.0
Weaving section		
Used	109	76.0
Not used	34	24.0
Finishing section		
Used	65	87.6
Not used	9	12.4
Engineering section		
Used	57	87.3
Not used	8	12.7
Garment section		
Used	94	88.2
Not used	12	11.8

More than 17.0 % of the workers reported that they did not use all the necessary PPE during work. The reasons for not using PPE were for 43.0 % lack of PPE, for 20.0 % lack of practice, for 20 % uncomfortable to use, and for 17.0 % lack of safety education.

### Factors associated with PPE utilization

Table 4 presents factors which remained statistically significant in the bivariate and multivariate logistic regression analyses. In this study, the independent predictors of PPE utilization on the multivariate analysis include service duration of >10 years [AOR: 0.23, 95 % CI: (0.09, 0.58)], availability of PPE [AOR: 21.73, 95 % CI: (8.62, 54.79)], shift work [AOR: 2.28, 95 % CI: (1.12, 4.66)], alcohol drinking [AOR: 0.26, 95 % CI: (0.10, 0.66)], and cigarette smoking [AOR: 0.20, 95 % CI: (0.05, 0.78)], (Table 4).

**Table 4:** Factors associated with associated with PPE utilization among textile factory workers in Hawassa Town, southern Ethiopia, 2014

Variables	Utilized PPE		Crude OR (95 % CI)	Adjusted OR (95 % CI)
	Yes	No		
Service duration (in year)				
1–10	272	78	1.0	1.0
> 10	272	38	2.05 (1.35, 3.13)	0.23 (0.09, 0.58)
PPE available				
Yes	531	62	0.03 (0.02, 0.05)	21.73 (8.62, 54.79)
No	13	54	1.0	1.0
Shift work				
Yes	475	87	0.44 (0.27, 0.71)	2.28 (1.12, 4.66)
No	69	29	1.0	1.0
Drink alcohol				
Yes	106	24	1.08 (0.66, 1.77)	0.26(0.10, 0.66)
No	438	92	1.0	1.0
Smoke cigarette				
Yes	86	7	0.34 (0.15, 0.76)	0.20 (0.05, 0.78)
No	458	109	1.0	1.0

## Discussion

In this study, the magnitude of PPE utilization among textile factory workers was 82.4 %. This finding is by far higher than that of studies from Asia (12.0–49.4 %) [14, 20] and Africa (16.7–75.3 %) [12, 13, 15, 18, 21]. The difference could be due to methodological differences, like study population and methods of data collection, and workplace conditions, like employees’ level of awareness on hazard control and disease prevention and accessibility to safety services. However, this does not mean that there will be no need for further strengthening the safety programs as there are significant proportion of the workers still does not use all the necessary PPE during work. The interventions should consider designing and provision of PPE, and regular safety education.

One important finding of this study was the identification of independent predictors influencing PPE utilization. It was found that the odds of PPE utilization among workers who served for greater than 10 years were 0.77 times less when compared to those who served for less than or equal to 10 years. The possible explanation for this may be that those who served for longer period could be accustomed to the work environment and developed false consciousness of safety which might drive them not to comply with safety precautions including proper use of PPE. Thus, giving attention for this segment of workers may contribute for safety improvement.

Like in previous studies [16, 18], availability of PPE was found to be strong independent predictor of PPE utilization in this study. Cognizant of this fact, safety administration programs should consider availing of all the necessary PPE on regular bases to promote health and safety in the workplace.

In this study the odds of PPE utilization among workers who worked in shift were about two times high when compared to those who did not. The reason behind this could be that shift work encourages workers towards PPE utilization by providing adequate time to think for and communicate about their safety. This signifies that there is a need for revising of work schedule to promote use of PPE in the factory.

Cigarette smoking and alcohol drinking also showed significance association with PPE utilization. The odds of PPE utilization among cigarette smokers and alcoholists were 0.80 and 0.74 times less, respectively when compared to their counter parts. This might be due to the fact that smoking and drinking are proxy indicators of a certain degree of risk tolerance. A high blood level of such substances during work will endanger both safety and efficiency, and be the cause of increased likelihood of mistakes, poor decision making, and errors in judgment. As the result of this fact, the factory’s safety policy should consider control of substance abuse at workplace.

Social desirability bias is a potential limitation in self–reported studies like this one, in that workers might report socially acceptable responses than their actual day to day practice. As this is a cross–sectional study, the limitations that come with this type of design need to be taken into consideration when interpreting the findings.

## Conclusion

In this study a relatively higher PPE utilization rate was observed compared to other studies in developing countries. However, this does not mean that there will be no need for further strengthening the safety programs as there are significant proportion of the workers still does not use all the necessary PPE during work. Interventions to promote PPE utilization should focus on areas, such as service duration, availability of PPE, presence of shift work, and control of substance abuse.
